# First-Pass Contrast-Enhanced Myocardial Perfusion MRI in Mice on a 3-T Clinical MR Scanner

**DOI:** 10.1002/mrm.22470

**Published:** 2010-10-06

**Authors:** Marcus Makowski, Christian Jansen, Ian Webb, Amedeo Chiribiri, Eike Nagel, Rene Botnar, Sebastian Kozerke, Sven Plein

**Affiliations:** 1 Division of Imaging Sciences, The Rayne Institute, King's College LondonSt Thomas' Campus, London, United Kingdom; 2 The Cardiovascular Division, Kings College London, The Rayne Institute, St Thomas' HospitalLondon, United Kingdom; 3 Institute for Biomedical Engineering, University and ETHZurich, Switzerland; 4 Division of Cardiovascular and Neuronal Remodelling, University of LeedsLeeds, United Kingdom

**Keywords:** MRI, Myocardial Perfusion, 3-T, Gadopentetate Dimeglumine, small animal

## Abstract

First-pass contrast-enhanced myocardial perfusion MRI in rodents has so far not been possible due to the temporal and spatial resolution requirements. We developed a new first-pass perfusion MR method for rodent imaging on a clinical 3.0-T scanner (Philips Healthcare, Best, The Netherlands) that employed 10-fold *k*-space and time domain undersampling with constrained image reconstruction, using temporal basis sets (*k-t* principle component analysis) to achieve a spatial resolution of 0.2 × 0.2 × 1.5mm^3^ and an acquisition window of 43 msec. The method was successfully tested in five healthy and four infarcted mice (C57BL/6J) at heart rates of 495.1 ± 45.8 beats/min. Signal-intensity-time profiles showed a percentage myocardial signal increase of 141.3 ± 38.9% in normal mice, compared with 44.7 ± 32.4% in infarcted segments. Mean myocardial blood flow by Fermi function for constrained deconvolution in control mice was 7.3 ± 1.5 mL/g/min, comparable to published literature, with no significant differences between three myocardial segments. In infarcted segments, myocardial blood flow was significantly reduced to 1.2 ± 0.8 mL/g/min (*P* < 0.01). This is the first report of first-pass myocardial perfusion MR in a mouse model on a clinical 3-T MR scanner and using a *k-t* undersampling method. Data were acquired on a 3-T scanner, using an approach similar to clinical acquisition protocols, thus facilitating translation of imaging findings between rodent and human studies. Magn Reson Med, 2010. © 2010 Wiley-Liss, Inc.

Genetically modified mice are increasingly used to study the pathophysiology of cardiovascular disease (1–4). One of the most relevant readouts in cardiovascular disease is myocardial blood flow (MBF), which is altered in several disease processes, including atherosclerosis, systemic hypertension, and diabetes mellitus (5–7). Therefore, accurate noninvasive assessment of MBF in rodent models of cardiovascular disease is highly desirable. Currently, such measurements may be obtained in rodents with nuclear perfusion imaging, echocardiography, microspheres, and spin labeling MRI (8–12). However, nuclear and echocardiographic perfusion methods are limited by relatively low spatial resolution. Microspheres prohibit serial assessment from the same animal, and concerns have been raised about the validity of microsphere measurements in rodents (13). In principle, MR offers high spatial resolution and tissue contrast and is therefore an attractive method for rodent imaging. Compared with larger animal models or human imaging, however, rodent MR is challenging because of the very high heart rates that are encountered (up to 600 beats/min) and the required high spatial resolution of ∼0.2mm voxel width in-plane. Spin labeling MR methods have been proposed for myocardial perfusion imaging in rodents, but these techniques require acquisition times of ∼25 min for a single slice, thus making it difficult to incorporate them in more complex imaging protocols (10).

In humans, the preferred method for myocardial perfusion assessment is dynamic first-pass contrast-enhanced acquisition. The method has been well validated in clinical models (7). However, first-pass perfusion assessment requires acquisition of a complete image at each or every second heartbeat and within a short acquisition window, following a magnetization preparation pulse. At the heart rates and required spatial resolution in rodents, first-pass contrast-enhanced perfusion MRI has been considered not feasible with conventional acquisition methods.

Over the past several years, methods that exploit spatiotemporal data correlations such as *k*-space and time domain undersampling [*k*-*t* sensitivity encoding (*k*-*t* SENSE)] have been developed, achieving acceleration of data acquisition by up to a factor of 8 in clinical studies (14). Further refinements of the original method, constrained image reconstruction using temporal basis sets [*k*-*t* principle component analysis (*k*-*t* PCA)] permit even higher acceleration factors, with fewer training profiles and improved temporal fidelity (15). With these methods, it may become feasible to perform first-pass contrast-enhanced myocardial perfusion imaging in rodents. The purpose of this study was to develop and show feasibility of first-pass contrast-enhanced myocardial perfusion cardiovascular magnetic resonance (CMR) in a rodent model at 3.0 T.

## MATERIALS AND METHODS

### Pulse Sequence Design

The myocardial perfusion method was implemented on a clinical 3.0-T system (Achieva; Philips Healthcare, Best, The Netherlands). The pulse sequence was based on a method previously used for human application (14) and adapted to the required field of view and temporal resolution in mice. A custom-written software patch allowed triggering of the scanner to a heart rate of up to 600 beats/min. Pulse sequence parameters were as follows: saturation recovery gradient echo mode, pulse repetition time/echo time 6.7 msec/1.0 msec, flip angle 20°, 10-fold undersampling with three training profiles, partial Fourier acquisition, one slice acquired during each RR interval, field of view 25 × 25mm^2^, slice thickness 1.5mm, matrix 128 × 128, spatial resolution 0.2 × 0.2mm^2^ (reconstructed to 0.13 × 0.13mm^2^), preparation pulse delay 100 msec, acquisition window 43 msec). To improve temporal fidelity of the data at the high undersampling factors used, the *k-t* PCA method was used for image reconstruction (15).

### Mouse Imaging

#### Animal Model

Nine 10- to 12-week-old homozygous C57BL/6J male mice were acquired from Charles Rivers Laboratories (Edinburgh, UK) or were bred within the Animal Unit of the Rayne Institute at King's College London. The housing and care of the animals and all the procedures used in these studies were performed in accordance with the guidelines and regulations of the United Kingdom Home Office. Five animals served as healthy controls, and in four animals permanent myocardial infarction was induced 3 days prior to imaging. For the induction of myocardial infarctions, mice were anesthetized by isoflurane inhalation and ventilated by tracheal intubation. Following left-side lateral thoracotomy, an 8-0 nylon suture was carefully placed around the left anterior descending coronary artery and was tied by the edge of the left atrium.

After the completion of the imaging study, 300 μL of Evans Blue (2% w/v) was injected into the tail vein to delineate the area at risk and the animals were sacrificed. Hearts were excised, mounted in agarose, and sliced into 1.5mm sections from apex to base. Slices were fixed overnight in 10% formaldehyde and were then digitally imaged, using a high-resolution optical scanner. Areas stained negative for Evans Blue were defined as not perfused and thus to be perfusion territory of the ligated coronary artery. Locations were identified as septal, anterolateral, and inferolateral, as described for perfusion MRI. Areas stained positive for Evans Blue were considered remote zones.

#### Preparation and Sedation

**FIG. 1:**
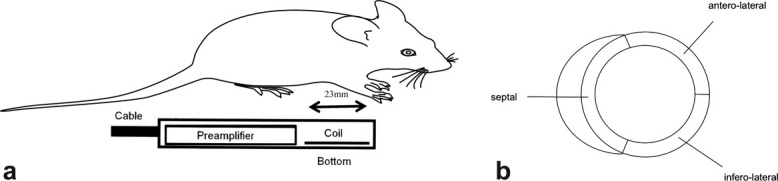
a: Design and dimensions of the single circular loop microscopy coil with respect to mice used for myocardial perfusion imaging. b: Diagram of the three-segment model used for analysis of MBF in mice. The myocardium was divided into three equidistant sectors of equal circumferential extent along the myocardial centerline, starting from a reference point placed at the anterior septal insertion of the right ventricle.

Mice were anesthetized and maintained under inhalational anesthesia via nose cone (1.4% isoflurane/oxygen). Rectal temperature was monitored continuously and a warm air flow (using an MR-compatible heater system; SA Instruments, Stony Brook, NY) was adapted to maintain temperature at 37°C. For reliable electrocardiogram (ECG) synchronization and artifact-free high-resolution imaging, a dedicated small-animal ECG device, 1025-MR (SA Instruments), and dedicated microscopy receive coils (23mm diameter, single circular loop; Philips Healthcare) were used (Fig. 1a). Two ECG leads were placed subcutaneously on the left and right side of the thorax.

#### MRI

All data were acquired during free breathing of the animal. For localization of the heart, a low-resolution gradient echo scout scan was acquired in the coronal and transverse orientations, using the following parameters: field of view 300 × 300mm^2^, matrix 300 × 300, slice thickness 2mm, pulse repetition time/echo time 20/5.8 msec, flip angle 20°, number of slices nine. Subsequently, pseudo two- and four-chamber gradient echo cine scans were acquired with prospective ECG-triggered spoiled gradient echo sequences. Imaging parameters were field of view 70 × 70mm, matrix 224, slice thickness 1.5mm, pulse repetition time/echo time 14/5.3 msec, flip angle 30°, two lines/RR interval, temporal resolution 18 msec. High-resolution (0.2 × 0.2 × 1mm^3^) short-axis, two- and four- chamber (biplane) views were then planned on those views. Imaging parameters were field of view 35 × 35mm^2^, matrix 160, slice thickness 1mm, pulse repetition time/echo time 11/5.3 msec, flip angle 20°, one line/RR interval, temporal resolution 11 msec, number of signal averages two. These images allowed for planning of the true short-axis views of the left ventricle. Perfusion imaging was performed in midventricular short-axis orientation, using the pulse sequence described above.

#### Contrast Delivery

A catheter was placed in the tail vein of the mouse and 0.1 mmol/kg bodyweight Gadolinium Gadopentetate Dimeglumine (Gd-DTPA) (Magnevist, Schering, Germany) was injected after the perfusion scan was started. To ensure a reproducible bolus injection, the contrast agent, as well as 25 μL of saline, was preloaded into small-bore tetrafluoroethylene tubing and injected manually over 3 sec.

### Data Analysis

Semiquantitative analysis was performed using dedicated image analysis software (Mass 7.1, Medis; Leiden University, Leiden, The Netherlands). Endocardial and epicardial contours were drawn on images with best blood to myocardium contrast and copied to all other dynamic images. The position of individual contours was then manually corrected to account for respiratory motion. The myocardium was divided into three sectors of equal circumferential extent along the myocardial centerline, starting from a reference point placed at the anterior septal insertion of the right ventricle (Fig. 1b).

To obtain the arterial input function, a region of interest was drawn inside the left ventricular (LV) blood pool. Signal intensity (SI)/time curves were generated for the LV blood pool, for the myocardium as a whole, and for the three myocardial sectors. The maximal upslope of the profiles was generated using five-point fitting. SI profiles were then generated for each sector and the region of interest in the LV blood pool. Enhancement ratio of signal increase and normalized SI upslope ratios between the blood pool and myocardium were calculated as (enhancement ratio = (SI max – SI baseline)/SI baseline) and (normalized SI upslope = upslope myocardium/LV).

In addition to this semiquantitative upslope-based analysis, absolute MBF was computed from the LV blood pool and myocardial tissue SI vs. time curves, using Fermi constrained deconvolution (16,17). The deconvolution algorithm was implemented in-house in MATLAB® (The MathWorks, Inc., Novi, MI), for the analysis of the data derived from the Mass image analysis software. The curves were baseline corrected and cropped to the first pass only (16,17).

### Statistics

Continuous data were expressed as mean ± standard deviation. For comparing groups (>2), the analysis of variance test was used, and for comparing pairs of a group, Tukey's test was used. Statistical significance was considered for *P* < 0.05 and high significance was considered for *P* < 0.01.

## RESULTS

Myocardial infarcts were successfully induced in the four operated mice. In the midventricular slice that was investigated by perfusion imaging, all infarcts were localized in the anterolateral myocardial segment. All areas stained negative for Evans Blue matched with the MR perfusion defect.

First-pass myocardial perfusion imaging was successfully performed in all five control and the four infarcted mice. All mice tolerated the scanning without any complications. The observed heart rate was within the expected physiological range, with no significant hemodynamic effect from the anesthetic observed. There was no significant difference in heart rate between the two groups (control group: 485 ± 50 beats/min, infarcted group 509 ± 45 beats/min, *P* > 0.05).

**FIG. 2:**
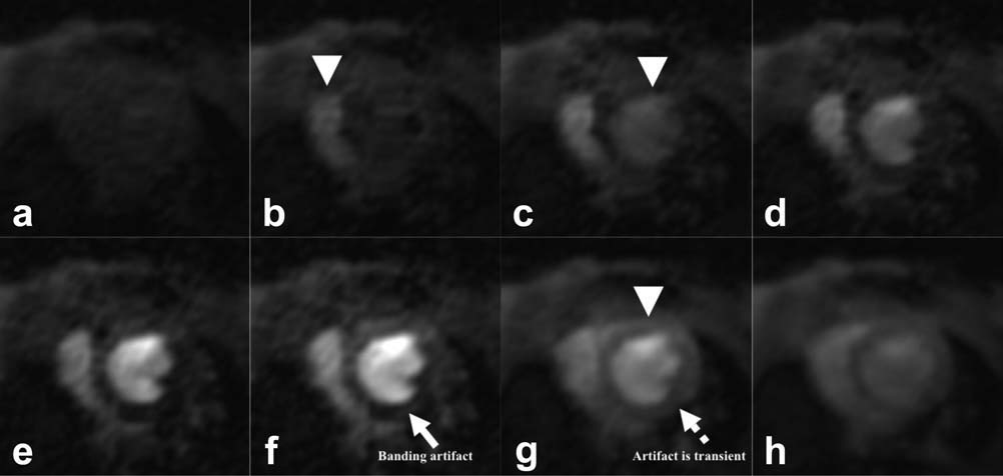
Series of dynamic images after contrast bolus injection (0.1 mmol/kg body weight Gd-DTPA) in one control mouse. Images demonstrate the baseline scan (a), as well as the passage of the contrast agent in the RV ((b), arrowhead), LV ((c), arrowhead), and gradually in the myocardium ((d–g), arrowhead). Note transient dark banding artifact in the inferior wall (arrows, (f,g)).

Typical transient subendocardial dark rim artifacts were noted in three animals, affecting in particular the inferolateral segments (Fig. 2). These banding artifacts persisted for three to seven heartbeats during peak signal in the LV and affected 30–60% of the myocardial thickness. However, artifacts did not affect semiquantitative and quantitative measurements.

### MBF Measurements

**FIG. 3:**
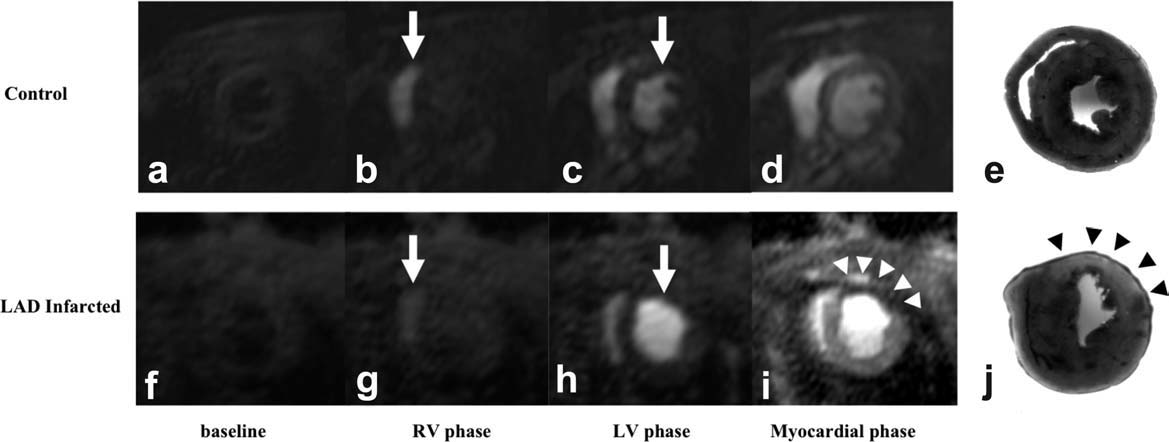
Series of key dynamic images after contrast bolus injection comparing one control (a–e) and one infarcted mouse (f–j). Image demonstrates the baseline (a,f) scans, the passage of the contrast agent in the RV (arrow, (b,g)), LV (arrow, (c,h)), and the myocardium (d,i). Note the attenuated signal in the anterior wall of the infarcted mouse (i). Images on the right (e,j) represent matched Evans Blue sections (2% w/v, injection into the tail vein) to delineate the area at risk. Note colocalization of the large anterior perfusion defect, using MRI with decreased staining in the matched Evans Blue section (i,j).

**FIG. 4:**
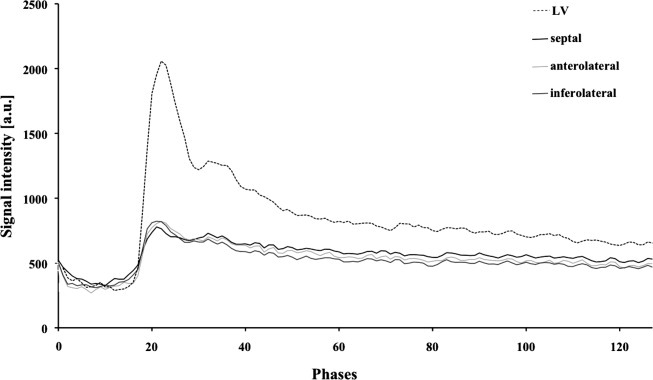
SI/phase profile from one control mouse. The dotted line represents the passage of the contrast agent (CA) in the LV cavity. The continuous lines represent the passage of the CA in the three different segments of the myocardium.

**FIG. 5:**
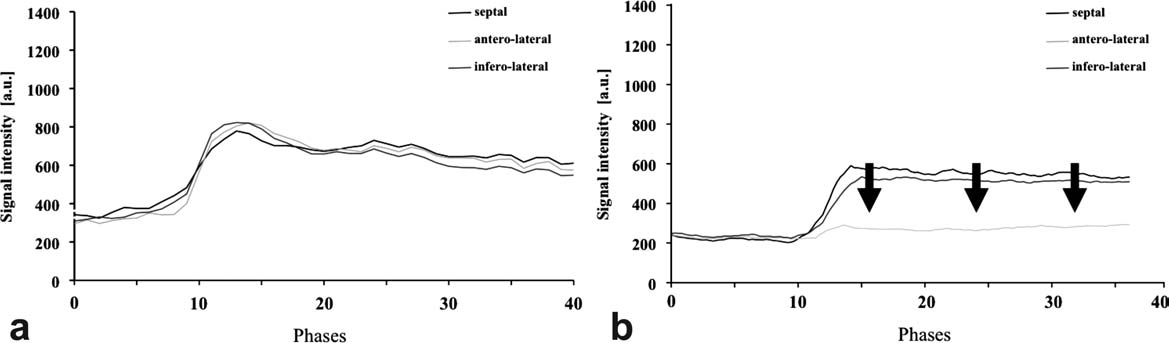
SI/phase profiles of one control (a) and one infarcted mouse (b) represent the passage of the CA in the myocardium (LV profile is not displayed). Note especially the light-grey line ((b), arrows), which represents the attenuated passage of the contrast agent in the anterolateral wall after myocardial infarction (anterolateral segments).

**FIG. 6:**
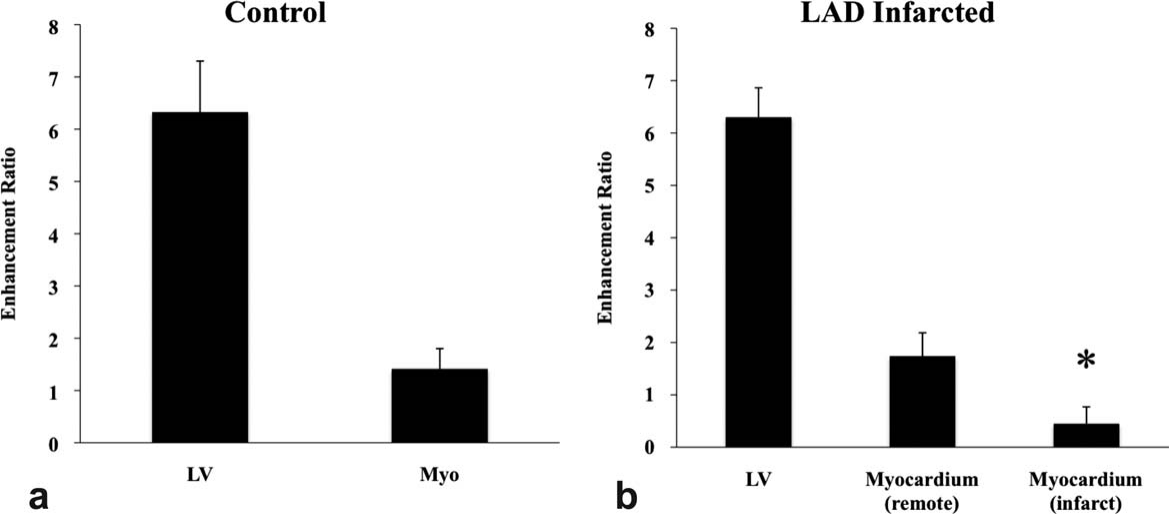
Graphical representation of the enhancement ratio in signal increase ((SI max – SI baseline)/SI baseline) averages and standard deviations in control mice, as well as in mice after myocardial infarction. A significantly lower enhancement ratio was found in the anterior segments of the mice after permanent occlusion of the left anterior descending (*P* < 0.05) compared with the remote myocardium.

SI/phase profiles derived from control and left anterior descending infarcted mice (Figs. 3–5) showed similar features to human profiles. In control mice, LV signal increased from 293.8 ± 69.1 before contrast delivery to a peak of 2148.8 ± 570.5, resulting in an enhancement ratio of 6.32 ± 0.98. Myocardial signal increased from 290.8 ± 63.3 to 694.8 ± 154.9, with an enhancement ratio of 1.41 ± 0.39 (Fig. 6). The normalized SI upslope in normal segments was 7.8 ± 2.3. The mean estimated MBF by Fermi-constrained deconvolution in all myocardial segments in control mice was 7.3 ± 1.5 mL/g/min. There were no significant differences between the three segments: septum (7.4 ± 1.7 mL/g/min), anterolateral wall (6.7 ± 1.5 mL/g/min), and inferolateral wall (7.8 ± 1.2 mL/g/min; *P* > 0.05).

**FIG. 7:**
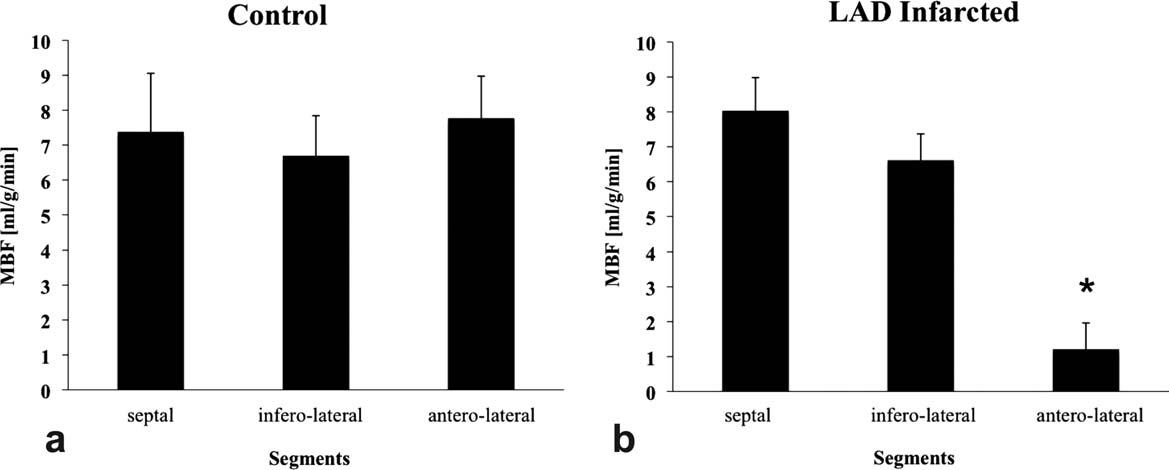
Graphical representation of regional MBF group averages and standard deviations in control and infarcted mice. No significant regional MBF variations could be found comparing the different segments in the control mice. However, a significantly lower MBF was found in the anterolateral segments of the infarcted mice (*, *P* < 0.05) compared with the inferolateral and septal segments.

In all infarcted mice, perfusion defects due to the myocardial infarction were localized in the anterolateral segment, matching the infarct location on histology (see Fig. 3). In infarcted mice, an enhancement ratio of 6.30 ± 0.56 (SI baseline: 380.8 ± 59.8; SI max: 2754.5 ± 215.3) was measured in the LV. In remote myocardial segments, the enhancement ratio was 1.74 ± 0.64 (SI baseline: 359.0 ± 147.9; SI max: 969.5 ± 44.7), while in the infarcted anterolateral segments, the mean enhancement ratio was only 0.45 ± 0.32 (*P* < 0.01) (SI baseline: 376.0 ± 129.2; SI max: 543.5 ± 35.4; Fig. 6). The normalized SI upslope in remote segments was 6.2 ± 3.0 versus 2.9 ± 1.1 in infarcted segments (*P* < 0.01). Estimated MBF differed significantly between normal and infarct segments (7.1 ± 1.5 vs. 1.2 ± 0.8 mL/g/min; *P* < 0.01; Fig. 7).

## DISCUSSION

This study is the first full report of first-pass contrast-enhanced MR myocardial perfusion imaging in mice. A previous account of bolus perfusion in mice on a small-bore, high-field MR system has recently been published in a conference proceeding (18). The present work is, to our knowledge, the first report of first-pass myocardial perfusion CMR in a mouse model on a clinical MR scanner and using a *k-t* undersampling method. The proposed method offers several potential improvements over previously used spin-labeling MR methods for myocardial perfusion imaging in rodents, in particular, faster data acquisition and closer correlation with clinically used imaging protocols.

The first-pass contrast-enhanced myocardial perfusion imaging protocol used in this study was based on highly accelerated data acquisition with the *k-t* PCA method. Thereby, high spatial resolution (0.2mm in-plane) and high temporal resolution (43 msec) imaging with accurate depiction of signal enhancements in the blood pools and in the myocardium was possible. Relative to previous *k-t* SENSE protocols for human application (10), a higher acceleration factor (10 vs. 5–8) was necessary and a lower number of training profiles had to be used to achieve the required acquisition speed. The *k-t* PCA framework for image reconstruction has been shown to improve temporal fidelity and thus permit robust measurements of myocardial perfusion at very high acceleration factors and with very few training profiles (15). In the present study, it also proved robust to respiratory motion, and despite the free-breathing experimental setup no apparent image artifacts relating to respiration were observed. In *k-t* PCA, temporal basis functions are derived based on the low-resolution training data acquired in every heartbeat. These basis functions will reflect significant respiratory-related bulk motion. Accordingly, image artifacts from respiratory motion are considerably reduced compared with *k-t* Broad-use Linear Acquisition Speed-up Technique (BLAST) and *k-t* SENSE reconstructions. Moreover, respiratory excursions of the anesthetized animals were shallow and relatively regular, which may have helped to minimize artifacts.

In this study, signal-time intensity profiles did not show signs of temporal low-pass filtering, and the calculated MBFs were comparable to that of published literature from arterial spin labeling and microsphere data (8–10,19). Infarcted segments demonstrated significantly reduced perfusion values, as expected.

Data in this study were acquired on a clinical 3-T CMR scanner, using an approach that closely resembles clinical acquisition protocols and thus facilitating clinical translation of imaging findings. Increasingly, rodent imaging is performed on clinical CMR systems (20) because these systems offer sophisticated acquisition protocols and off-the-shelf quantitative postprocessing methods, but field strength and gradient performance are lower compared with that of small-bore high-field magnets. High-field (> 4.7 T) small-animal systems provide the advantage of increased signal-to-noise ratio and spatial and temporal resolution, but the limited availability of novel acquisition and reconstruction methods may be a drawback. The previous conference report of myocardial perfusion on a dedicated 7-T high-field system yielded spatial and similar temporal resolution identical to that inour setup (18). However, findings made on dedicated preclinical systems may be difficult to translate into clinical practice due to the differences in field strength and system specifics, a limitation that applies much less if rodent scans are performed on clinical scanners.

Cardiovascular MR has been shown to hold potential for a comprehensive workup of cardiac function in rodents. Global and regional cardiac function can be quantified accurately with cine and tagging MR methods (4,21). Presence and extent of myocardial scar can be visualized using late gadolinium-enhanced MR acquisition (22,23). In addition, imaging of metabolic and molecular processes in heart disease is an emerging field and tremendous progress has been made in the last decade (24,25). The possibility to assess myocardial perfusion would add an important additional readout. Contrast-enhanced myocardial perfusion MR integrates well into such protocols because data acquisition is fast and the administered contrast agent is used for subsequent late gadolinium-enhanced imaging.

### Limitations

Contrast delivery in this study was manual but can in principle be automated to improve reproducibility. The optimal contrast dose and injection speed need to be optimized to account for physiological differences between rodents and humans. The current implementation only allows for the acquisition of one slice at each RR interval. More slices could potentially be acquired if acquisitions were shared between multiple RR intervals at the expense of lower temporal resolution or with further refinements of the acquisition protocol. Finally, the ability to acquire first-pass myocardial perfusion MR data during vasodilator stress needs to be tested and validated.

## CONCLUSIONS

We present a novel approach for first-pass myocardial perfusion MRI in rodents that provides MBF values within the expected range in normal and left anterior descending infarcted mice. The method is fast and may permit data acquisition under pharmacological stress. With the increasing range of mouse models in cardiovascular disease, this method could provide a useful tool to systematically investigate the role of ischemia in atherosclerosis, diabetes, hypertension, or heart failure.
